# Network‐Based Integrative Analysis to Identify Key Genes and Corresponding Reporter Biomolecules for Triple‐Negative Breast Cancer

**DOI:** 10.1002/cam4.70674

**Published:** 2025-04-27

**Authors:** Pooja Singh, Rupesh Chaturvedi, Pallavi Somvanshi

**Affiliations:** ^1^ School of Computational & Sciences (SCIS) Jawaharlal Nehru University New Delhi India; ^2^ School of Biotechnology (SBT) Jawaharlal Nehru University New Delhi India

**Keywords:** cumulative survival analysis, key gene, reporter biomolecule, triple negative breast cancer

## Abstract

**Background:**

The malignant neoplasm of the TNBC is the leading cause of death among Indian women. Recent studies identified the global burden of TNBC affecting approximately more than 40 percent of all BC cases in women worldwide. The absence of expression of receptors such as ER, PR, and HER2 characterizes TNBC.

**Objectives:**

Due to the lack of specific targets, standard treatment options for TNBC are limited. This integrative study aims to identify key genes and provide insights into the underlying molecular mechanisms of TNBC, which can potentially lead to the development of more effective therapeutic strategies.

**Material and Methodology:**

This study integrates PPI and WGCNA analysis of TNBC‐related datasets (GSE52194 and GSE58135) to identify key genes. Subsequently, downstream analysis is conducted to explore potential therapeutic targets for TNBC.

**Results:**

The present study renders the potential 13 key genes (PLCG2, CXCL10, CDK1, STAT1, IL6, PLK1, CCNB1, AURKA, NDC80, EGFR, 1L1B, FN1, BUB1B), along with their associated 6 TFs and 20 miRNAs, as reporter biomolecules around which the most significant changes occur. There were some miRNAs hsa‐mir‐449b‐5p, hsa‐let‐7b‐5p, hsa‐mir‐26a‐5p, hsa‐mir‐155‐5p, hsa‐mir‐24‐3p, hsa‐mir‐212‐3p, hsa‐mir‐21‐5p, hsa‐mir‐210‐3p and hsa‐mir‐20a‐5p whose association with other cancers and other BC subtypes have been reported but their association with TNBC need to be explored. Further, enrichment and cumulative survival analysis support the disease association of identified key genes with TNBC.

**Conclusion:**

This integrative analysis could be regarded for experimental inspection as it provides the platform for future researchers in drug designing and biomarker discovery for TNBC diagnosis and treatment.

## 1 Introduction

Breast cancer (BC) accounting for 12% of all prevailing cancers worldwide is a serious concern for public health globally [1]. Due to its heterogenous nature, BC is categorized into three main groups based on cellular receptor markers reflecting available targeted therapies: (a) estrogen receptor (ER) or progesterone receptor (PR) positive; (b) human epidermal growth factor receptor 2 (HER2) positive (amplification of erbB2) with or without ER and PR positivity; and (c) triple‐negative breast cancer (TNBC) defined by the absence of all kinds of receptor markers expression like ER, PR, and HER2 [2]. Due to the advancement of genomics technologies and proper management by government authorities, major contributing factors responsible for TNBC surveillance and prevention have been identified. Still, there are no standard treatment options available for TNBC because it does not respond to drugs that target receptors like ER, PR, and HER2, which accounts for 10%–20% of all invasive BC cases [2, 3]. Since TNBC is more likely to metastasize to the liver, bones, and lungs: it is usually diagnosed late and the survival period is short once it spreads. So, we really need new ways to spot it early [4]. Hence, there is an urgent need to find new biomarkers and their robust finding technique/pipeline facilitating the early‐stage detection of the disease. Biomarkers are generally classified into four main categories: diagnostic, prognostic, predictive, and therapeutic, each with distinct importance [5]. Diagnostic biomarkers have the potential to identify noninvasively the presence of disease, prognostic biomarkers provide information on patient survival with or without treatment, and predictive biomarkers help to determine which treatment is most likely to improve a patient's survival whereas therapeutic biomarkers, often proteins, serve as targets in treatment therapies [5].

Array‐based sequencing techniques, including microarrays and RNA‐Seq, are potential cutting‐edge high‐throughput genomic/transcriptomic sequencing methods. The microarray methodology is based on hybridization, whereas the RNA‐Seq method is based on synthesis and uses DNA polymerase to insert nucleotides [6]. Unlike arrays, RNA‐Seq technology does not require species‐ or transcript‐specific probes and may discover novel transcripts, gene fusions, single nucleotide variations, and indels (small insertions and deletions) [7, 8]. Recent studies have employed array and RNA‐seq data, on which bioinformatics approaches have been implemented to identify key/hub genes for TNBC [9, 10, 11, 12]. These studies have revealed that most of the identified key/hub genes are kinases. Cross‐platform data integration in RNA‐seq analysis involves combining data from multiple sources, technologies, or studies to improve the robustness and depth of biological insights [13]. This approach can overcome limitations posed by individual datasets, due to small sample sizes or platform‐specific biases, by leveraging diverse data to enhance statistical power and uncover broader patterns in expression profiling. In today's era of bioinformatics, collecting data is not the main challenge; instead, normalizing the data poses a significant hurdle [14]. Considering all these factors, we devised an integrated protein–protein interaction (PPI) and weighted gene co‐expression network analysis (WGCNA) study aimed at identifying crucial key genes linked to TNBC along with their associated TFs and miRNAs as reporter biomolecules [15, 16] around which the most significant changes occur, which could be used as a potential biomarker to cure the disease. Initially, we retrieved TNBC‐associated RNA‐Seq datasets from the GEO database. Before integrating the data, we conducted preprocessing and normalization procedures, following established methods documented in existing literature deemed suitable for our study. In this integrated analysis aimed at identifying key genes, the pipeline was bifurcated into two main parts. The first part involved identifying differentially expressed genes (DEGs) and subsequently reconstructing a PPI network to retrieve significant hub genes. This was followed by enrichment analysis, which supported the involvement of DEGs in cancer‐related pathways and biological ontologies. The second part focused on WGCNA analysis, where a co‐expression network was constructed to elucidate correlations between gene clusters and phenotypic attributes. Furthermore, phenotypically significant clusters were identified. Moreover, key hub genes were retrieved, and downstream analyses such as the exploration of associated regulatory biomolecules, cross‐validation, and novel cumulative survival analysis were conducted to establish them as potential biomarkers for TNBC. This novel cumulative survival method surpasses traditional survival analysis, which typically evaluates the prognostic power of individual genes over time but often fails to fully capture the underlying mechanisms of disease progression. This new approach can identify multiple gene targets within disrupted pathways, facilitating the development of more effective drugs to improve survival rates in the future.

## 2 Materials and Methodology

### 2.1 Data Retrieval

To achieve a comprehensive analysis, a thorough literature review was conducted to identify all the publicly available RNA‐seq datasets containing both normal and cancerous samples associated with TNBC. Keywords such as “TNBC,” “ *Homo sapiens* ,” and “expression profiling by high throughput sequencing” were used for the thorough literature search. Four unprocessed RNA‐seq transcriptome datasets (GSE52194, GSE58135, GSE142258, and GSE142731) were obtained from the literature available on the GEO database [17]. Of these, two datasets (GSE58135, GSE52194) were selected for evaluating integrative gene expression profiling in TNBC, as they included samples from both healthy and diseased individuals. The other two datasets (GSE142258 and GSE142731) were excluded due to the absence of normal samples, to maintain the homogeneity of analysis.

### 2.2 Quality‐Check and Data Integration

Using FastQC v0.11.5 toolkit [18] the quality of the unprocessed sequence data was examined. After the quality check, poor‐quality reads and adapters were cropped and trimmed using the tool Trimmomatic v0.36 [19]. After processing, reference‐based alignment was performed on the processed reads using the tool STAR 2.7.10a [20]. The human genome GRCh38. DNA (Ensembl release 107) was used as the reference, and default parameters in STAR for alignment were employed because these settings are optimized for mammalian genomes. Followed by this, FeatureCounts v1.6.2 [21] was used to quantify each read using the same Ensembl release (107) annotation file. Before the data integration, pre‐filtering was performed to remove low‐count genes and select rows with at least five reads within each dataset. Furthermore, before integrating the data using the merge() R function, normalization and GC BIAS correction were performed using the cqn() R package [22], and again pre‐filtering was performed on integrated datasets to remove low‐count genes, which were less than 10 in 75% of samples to make the data more consistent and robust for the further analysis.

### 2.3 Dataset Analysis

**FIGURE 1:**
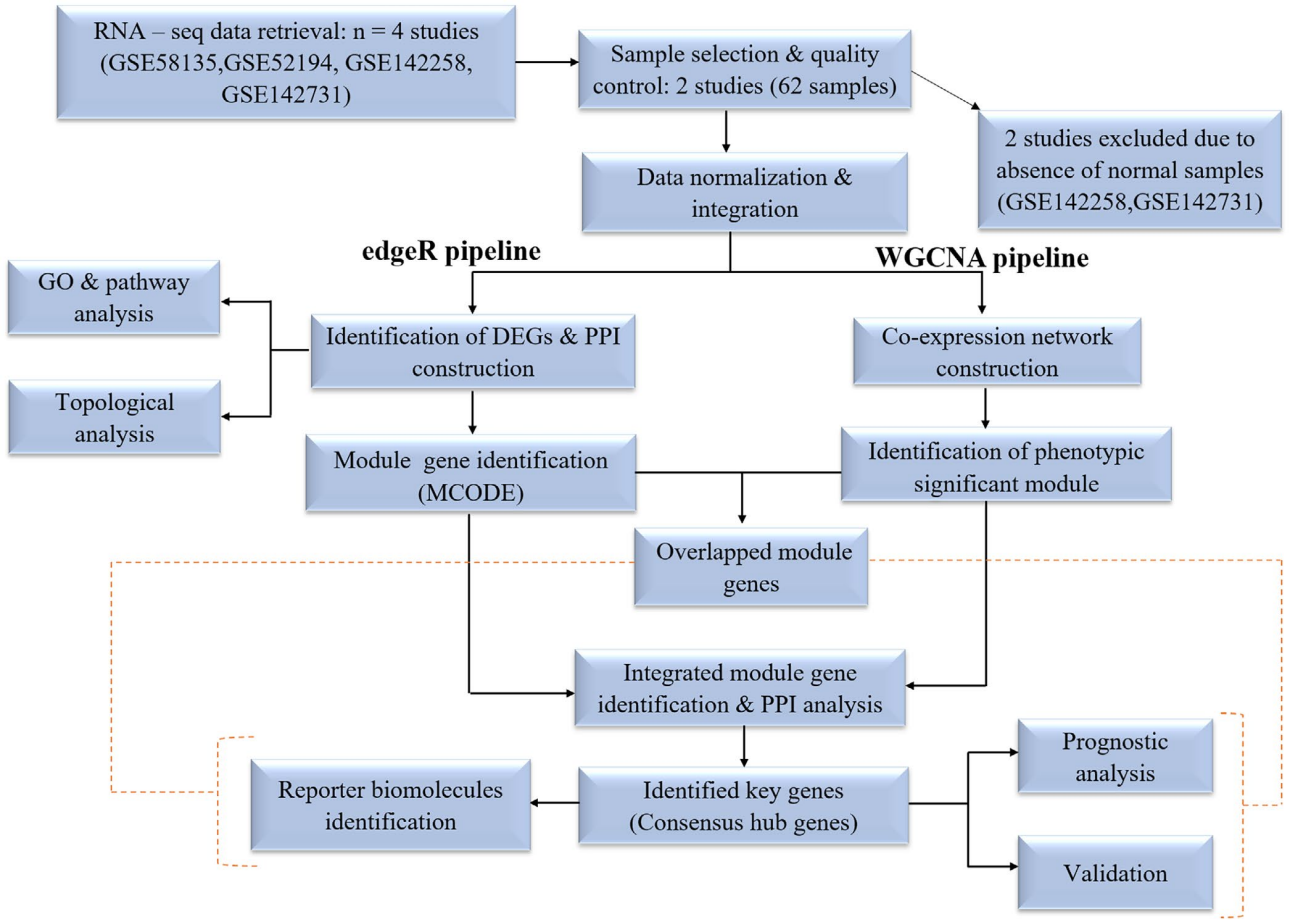
Overview of the study.

After integrating the data, the entire pipeline was bifurcated into two distinct categories as shown in Figure 1. The first category was dedicated to conducting the differential gene expression analysis utilizing the edgeR [23] package. Simultaneously, the second category focused on performing the WGCNA [24] using the R package.

### 2.4 DEG Identification, PPI Reconstruction and Module Identification

In the present study using the edgeR package, genes with log2FoldChange > 1.0 and adjusted *p* (*p* adj) < 0.01, corrected by the Benjamini‐Hochberg method, were considered upregulated or overexpressed, while log2FoldChange < −1.0 and *p* adj < 0.01 were considered downregulated or under‐expressed, and we are calling them DEGs. The STRING database [25] with a confidence score of 0.90 was employed to reconstruct the PPI network of DEGs. The visualization of this network was accomplished using CytoscapeF [26]. In the PPI network, an undirected graph was employed, where ‘V’ denoted a set of vertices representing nodes (proteins), and ‘E’ represented a set of edges signifying connections between the proteins. To identify the significant module of DEGs, the Cytoscape plugin MCODE was employed to identify the finest cluster within the network.

### 2.5 WGCNA Analysis

To create WGCNA of integrated 16,384 gene counts, we used the WGCNA R package. Primarily, we created an adjacency matrix to outline the correlation strength between the nodes.

An intermediate co‐expression similarity matrix was calculated first to calculate the adjacency matrix. The following equations were utilized to derive the similarity and adjacency matrix:

(1) $$S_{\mathrm{ij}}=\&a_{\mathrm{ij}}={s^{\beta }}_{\mathrm{ij}}$$

In these equations, *i* and *j* represent two distinct genes, whereas *xi* and *xj* denote their respective expression values. *S*_*ij*_ signifies Pearson's correlation coefficient, whereas *a*_*ij*_ denotes the magnitude of the connection between two genes. For this study, we selected a soft‐threshold power of *β* = 15 (scale‐free *R* ^2^ = 0.90), which determines the specificity and sensitivity of the pairwise connection strengths used to construct the adjacency matrix. Following this, we transformed the adjacency matrix into a topological overlap matrix (TOM). The TOM matrix serves as a method to quantitatively depict the similarity in nodes by evaluating the weighted correlation between two nodes and other nodes. Following this, hierarchical clustering was conducted to pinpoint significant modules.

### 2.6 Enrichment Analysis of DEGs

Enrichment analyses referring to molecular function, biological process, and cellular activity of DEGs were performed using the ShinyGO 0.80 [27] tool, and to explore involved biological pathways, Kyoto Encyclopedia of Genes and Genomes (KEGG) [28] was employed in the analysis. In this inspection, *p*‐values derived by hypergeometric distribution:

(2) p=∑k=nabkN−ba−kNa

Where, *n* represents the number of DE genes in the gene set. *N* denotes the total count of genes included in the analysis. *b* represents the counts of genes within the gene set. *a* signifies the number of DE genes within the gene set.

Equation (2) utilizes Fisher's exact test, which underwent correction through an enhanced Benjamini‐Hochberg method as the multiple testing correction technique. Gene‐set enrichment outcomes with an adjusted *p* < 0.05 were deemed statistically significant.

### 2.7 Key Gene and Regulatory Biomolecule Identification

To identify the key genes in this integrative analysis, we focused on the overlapping and integrative module genes derived from our bifurcated pipeline. We conducted topological analyses, followed by constructing a PPI network on module genes. To identify significant regulatory biomolecules—transcription factors (TFs) and miRNAs—that collectively control key genes at transcriptional and translational levels, we used the TRRUST v2 [29] and miRTarbase [30] databases through the miRNet 2.0 [31] platform. Only biomolecules with an adjusted *p* < 0.05 were considered significant.

### 2.8 Cumulative Survival Analysis

To gain insight into the survival value of identified key genes, independent array datasets for breast cancer (study ID: brca_metabric), which include 1981 patient's clinical information, were employed. At first, we clustered the key genes based on their expression values using of *K*‐means clustering algorithm in R and grouped them into five clusters, denoted as *k* = 5. The patient's groups were divided into low and high groups based on their mRNA expression levels, which correspond to their cluster. For each cluster, Kaplan–Meier (KM) plots were generated to visually compare survival outcomes, and clusters with log‐rank *p* < 0.05 were considered statistically significant. Subsequently, Cox proportional hazard regression analysis was also performed to assess the association between the survival time of patients and predictor variables. By combining Kaplan–Meier plots with Cox regression analysis, we ensured a comprehensive evaluation of the survival impact, where the former offered a visual representation of survival differences and the latter provided a quantitative hazard ratio.

## 3 Results

### 3.1 Identification of DEGs

To identify the participation of DEGs in the association of TNBC as the disease is heterogeneous and differs remarkably by the absence of receptor biomarkers, we selected two publicly available gene expression datasets associated with TNBC that contained both cancerous and normal samples (Table 1). To identify DEGs of integrated datasets, the edgeR pipeline is utilized. Among the DEGs, we found that there are 2595 up‐ and 2001 down‐regulated (Table 2) genes that were found statistically significant with adj *p* ≤ 0.01.

**TABLE 1:** Datasets (TNBC).

GEO ID	Platform ID	Number of cancerous samples	Number of normal samples	Library layout	References
GSE58135	GPL11154	35	18	Paired	Varley et al. 2014 [32]
GSE52194	GPL11154	6	3	Paired	Eswaran et al. 2015 [33]

**TABLE 2:** DEGs analysis.

Merged count matrix	UP regulated genes (LogFC ≥ 1 and adj *p* ≤ 0.01)	Down regulated genes (LogFC ≥ 1 and adj *p* ≤ 0.01)
16,384 counts, 61 samples	2595	2001

### 3.2 Enrichment Analysis

**FIGURE 2:**
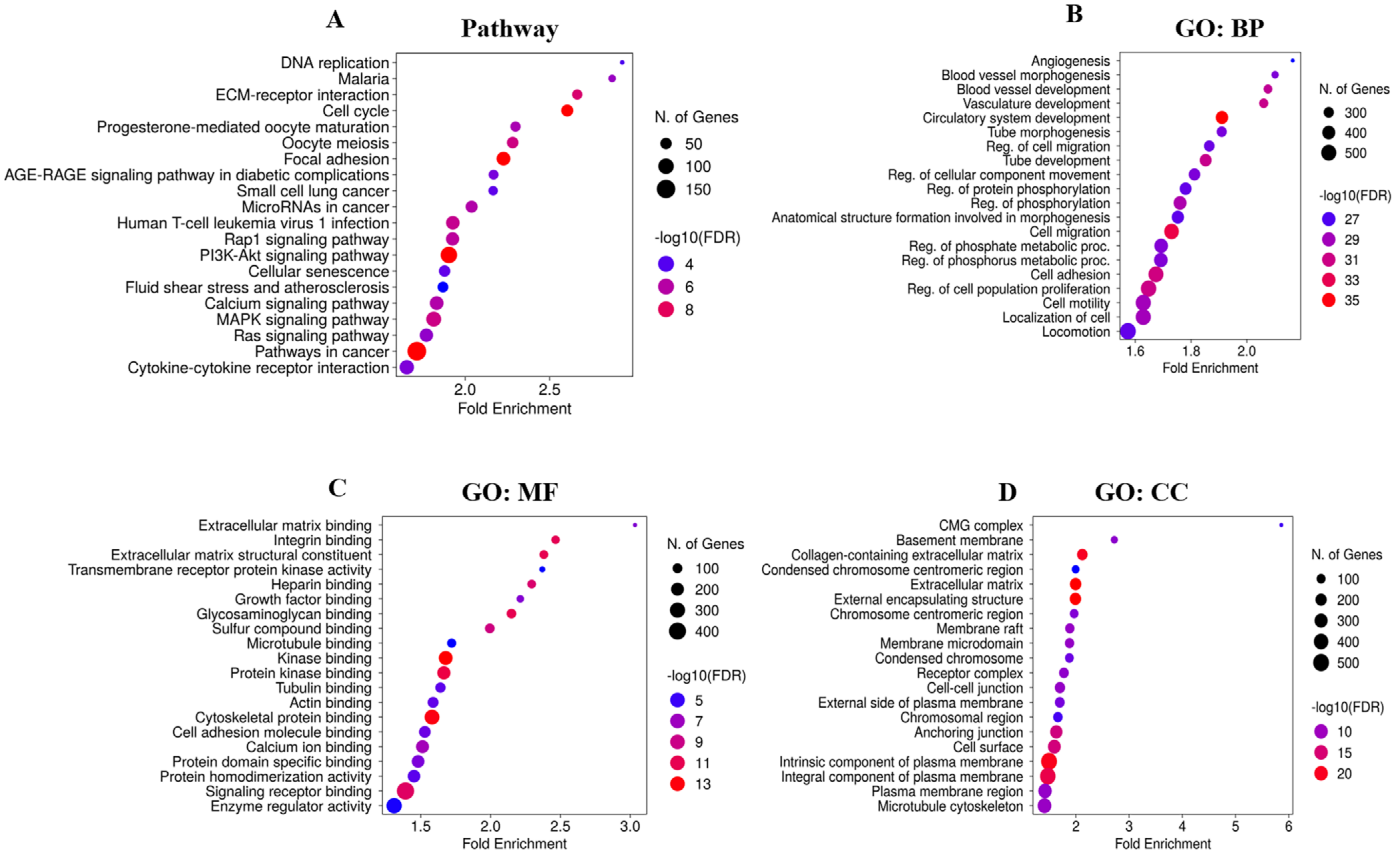
Enrichment analysis of differentially expressed genes (DEGs). (A) KEGG pathway, (B) Gene ontology biological process, (C) Molecular function, and (D) Cellular component analysis.

Pathway analysis revealed pathways in cancer, including the PI3K‐Akt signaling pathway, focal adhesion, cell cycle, MAPK signaling pathway, calcium signaling pathway, and other cancer pathways that were found to be influenced by DEGs (Figure 2A). GO ontology biological process reveals localization of cells, regulation of cell population proliferation, cell adhesion, cell migration, circulatory system development, and other processes associated with cancer development that were found to be influenced by DEGs (Figure 2B). Go‐term molecular function inspection uncovers enzyme regulator activity, signaling receptor binding, cytoskeletal protein binding, protein kinase binding, kinase binding, and calcium ion binding, which are the most molecular activities in which DEG involvement has been identified as statistically significant (Figure 2C). Whereas GO term cellular component analysis revealed the intrinsic component of the plasma membrane, integral component of the plasma membrane, plasma membrane region, extracellular matrix, external encapsulating structure, and cell surface, others were the cellular components affected by DEGs of TNBC (Figure 2D).

### 3.3 PPI Reconstruction of DEGs

**FIGURE 3:**
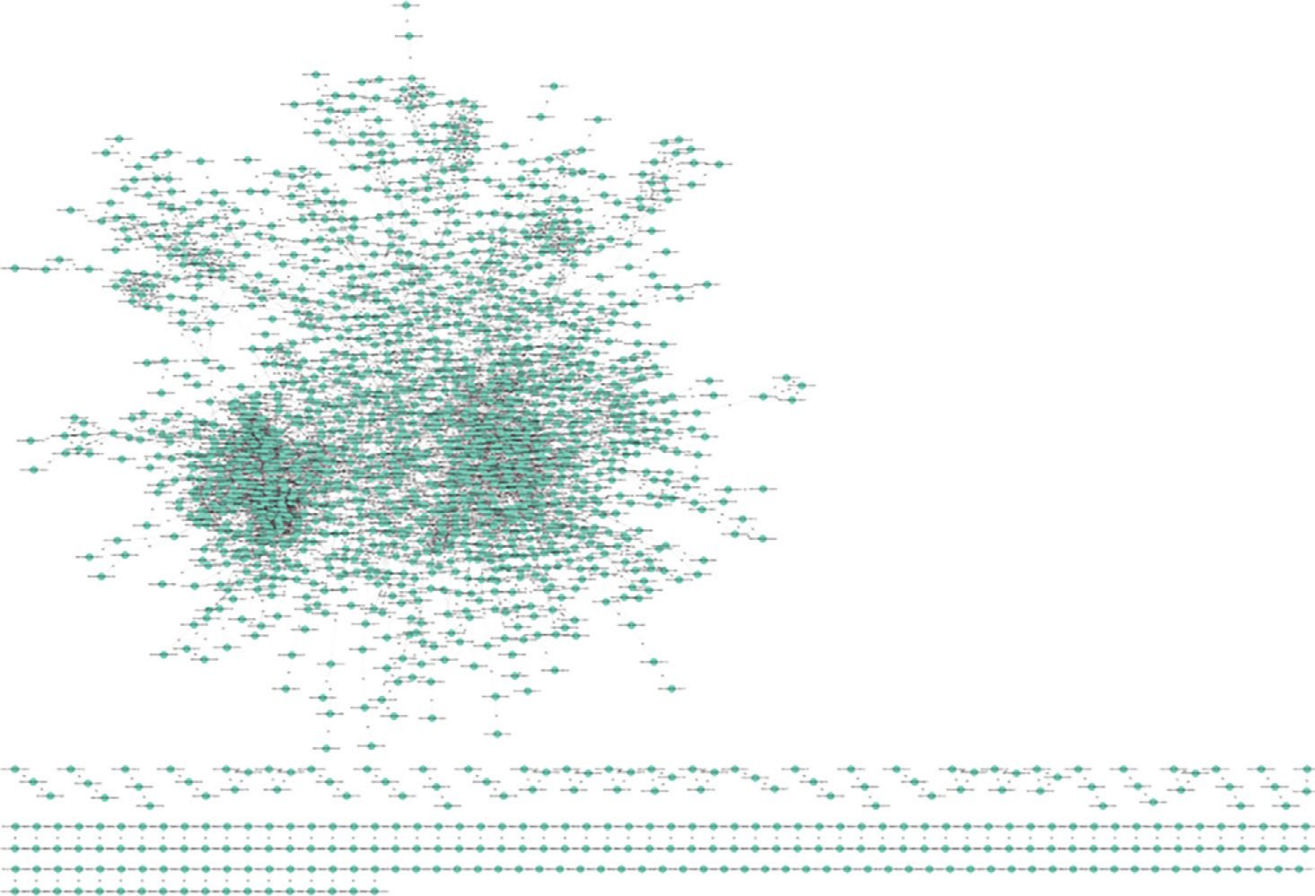
Reconstruction of protein–protein interaction (PPI) network for dysregulated genes of TNBC. A graph representation of PPI network for dysregulated genes using STRING database with confidence score > 0.90 involving 1961 nodes as protein and 6161 edges as interaction between them.

Further PPI networks for both up‐ and down‐regulated genes were reconstructed together. The PPI network consists of 1961 nodes as proteins and 6161 edges as interactions between them, demonstrating that the PPI network follows a scale‐free topology, where a few nodes have a higher degree of interaction with other nodes (Figure 3). The interconnections among the cluster genes within the entire network were identified using the Cytoscape plugin MCODE. There were seven clusters: 34 nodes and 489 edges in cluster1, 13 nodes and 78 edges in Cluster 2, 21 nodes and 125 edges in cluster 3, 12 nodes and 61 edges in Cluster 4, 32 nodes and 139 edges in cluster 5, 9 nodes and 35 edges in cluster 6, and 57 nodes and 210 edges in cluster 7, which were identified from MCODE based on a scoring system (cutoff *k*‐score ≥ 7) (Table 3).

**TABLE 3:** PPI module genes.

Module name	Score	No of nodes
Cluster1	29.636	34
Cluster2	13	13
Cluster3	12.5	21
Cluster4	11.091	12
Cluster5	8.968	32
Cluster6	8.75	9
Cluster7	7.5	57

### 3.4 WGCNA Phenotypic Significant Modules Identification

**FIGURE 4:**
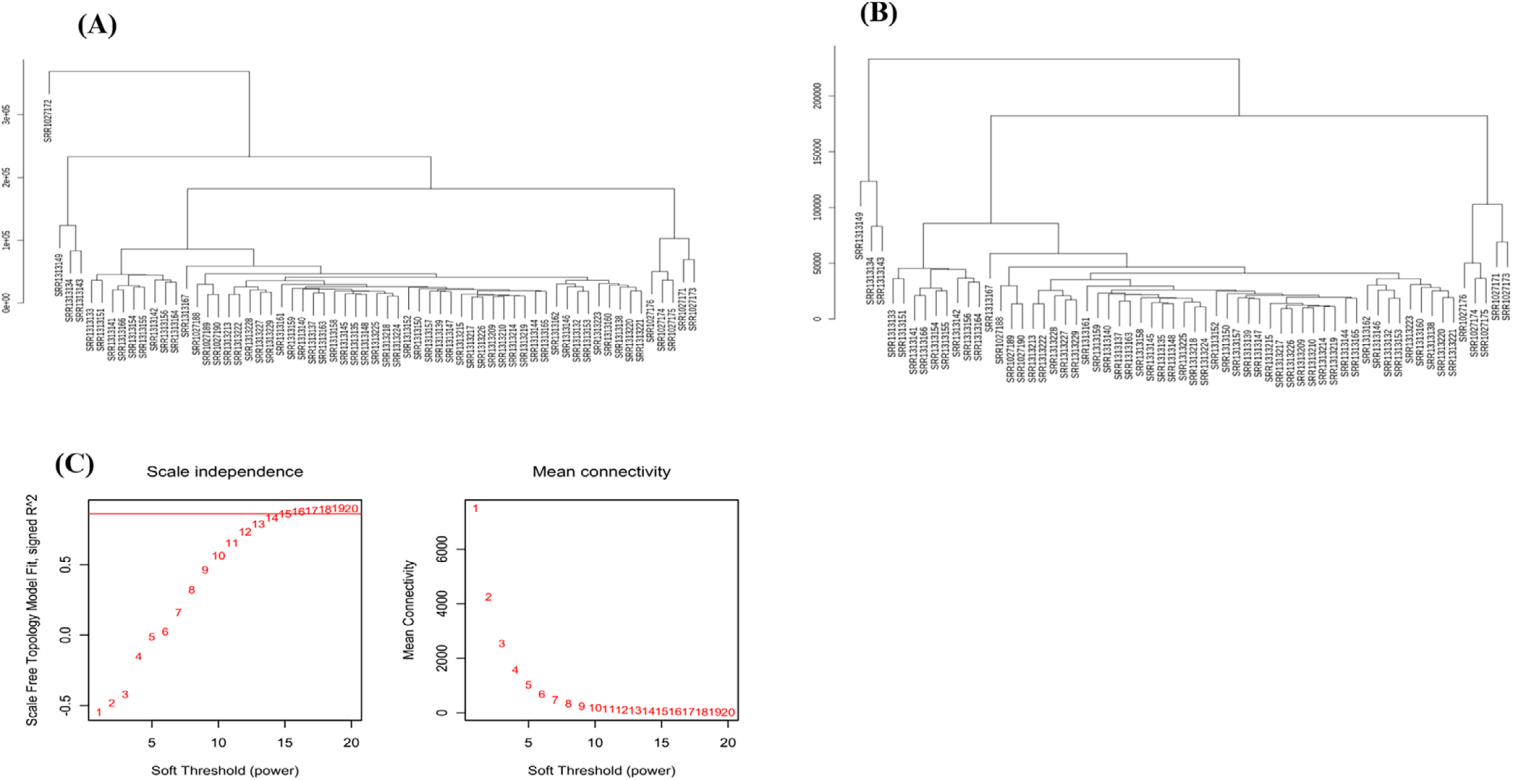
Preprocessing of weighted gene co‐expression network analysis and soft threshold (β power) selection to create adjacency matrix. (A) Sample clustering to detect outliers in selected samples of TNBC. (B) Processed samples. (C) The soft threshold selection, including the analysis of the scale‐free topology fitting the index *R* ^2^ (left) and the mean connectivity for the various soft threshold powers (right). In the left panel, the red line indicates, *β* = 15 chosen that corresponds *R* ^2^ = 0.90 for the model fitting.

**FIGURE 5:**
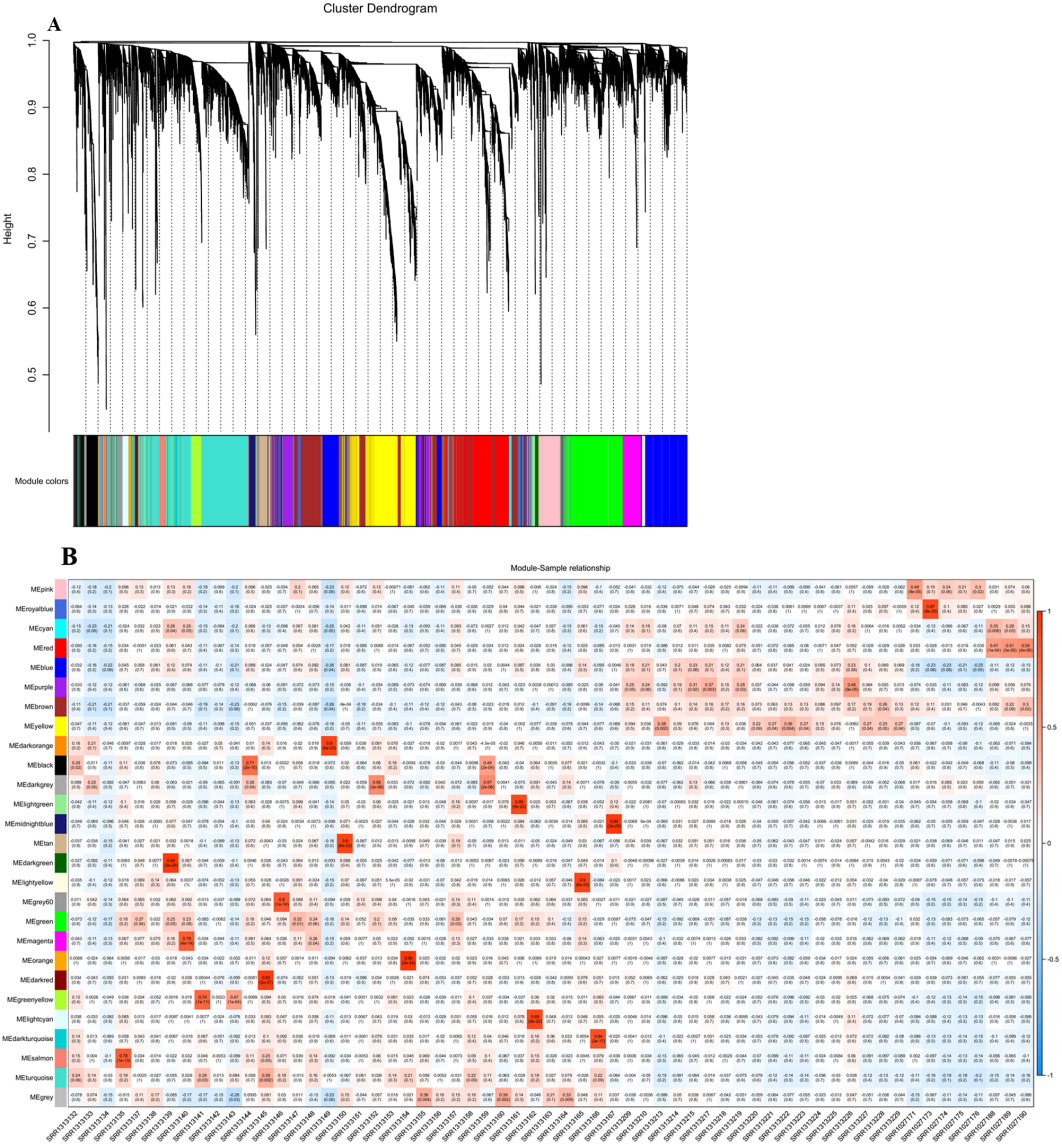
Weighted gene co‐expression network analysis. (A) Gene clustering tree built by hierarchical clustering of adjacency‐based dissimilarity to detect 27 co‐expression clusters with corresponding color assignments. (B) The module‐trait relationship. Each row correlates to module eigengene, column to trait (normal/cancer). Each cell includes the corresponding correlation and p‐value. Positive correlation is in red and negative correlation is in blue.

To create a co‐expression network of integrated 16,384 gene counts with the clinical trait cancer, primitively, preprocessing was conducted to identify outliers (Figure 4A,B) within the selected samples of TNBC using a clustering algorithm. A soft thresholding parameter of *β* = 15 (scale‐free *R* ^2^ = 0.90) was chosen to ensure a scale‐free network (Figure 4C). A dendrogram was created by clustering all the DEGs using a dissimilarity measure known as 1‐TOM. Through hierarchical clustering, 27 modules were identified (Figure 5A), among which 7 were found to have the highest association with cancer and were statistically significant (eigenvalue ≥ 0.90) (Figure 5B, Table 4).

**TABLE 4:** Phenotypic significant modules.

Module name	Phenotype	Correlation score	No. of genes
Dark green	Cancer	0.94	74
Dark orange	Cancer	0.90	55
Dark red	Cancer	0.93	80
Light yellow	Cancer	0.90	83
Midnight blue	Cancer	0.94	151
Orange	Cancer	0.96	63
Tan	Cancer	0.90	190

### 3.5 Key Gene Identification

**FIGURE 6:**
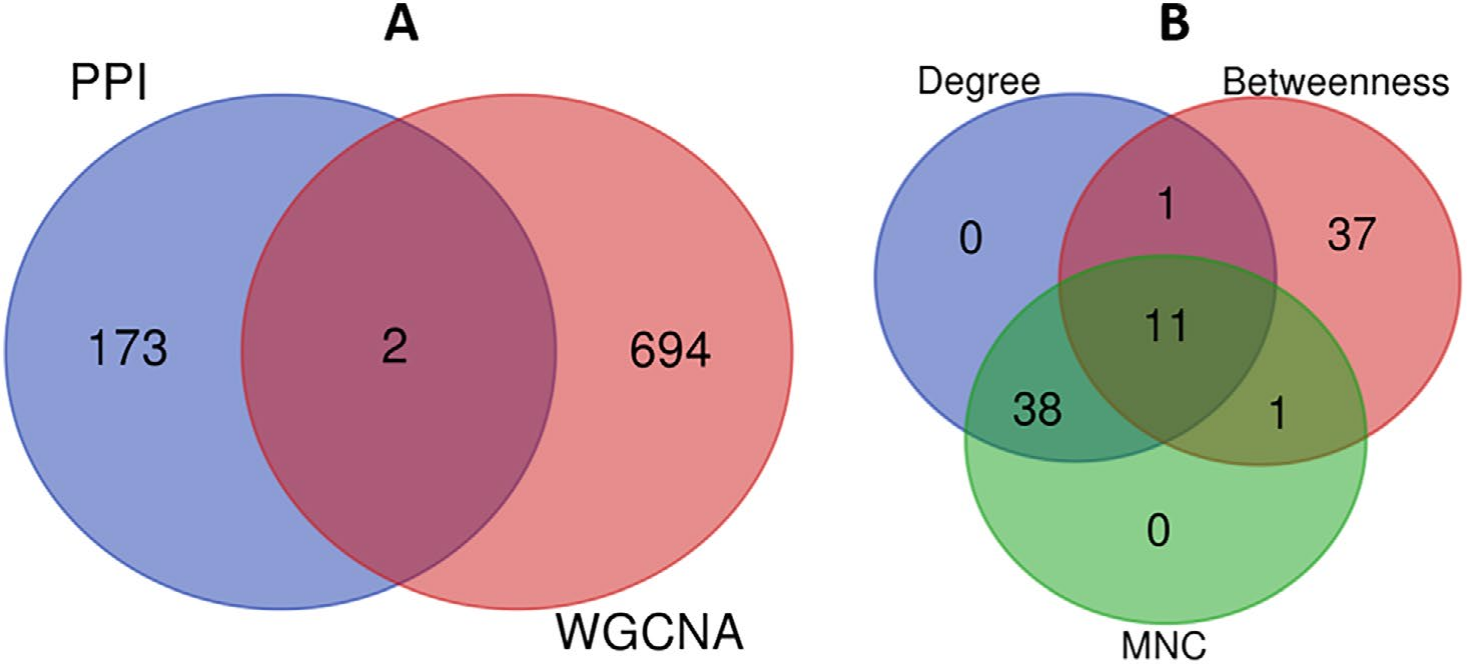
Venn diagram representation of consensus genes. (A) Two genes (PLCG2, CXCL10) found to be overlapped between module genes obtained from PPI analysis of dysregulated genes and WGCNA analysis. (B) Eleven genes have been identified as key genes, demonstrating consensus in terms of degree, betweenness, and maximum neighborhood component network topological properties.

When we took consensus between module genes identified through PPI and WGCNA, there are two genes, PLCG2 and CXCL10 (Figure 6A), and when we did integrative PPI analysis followed by topological analysis, 11 genes (Figure 6B, Table 5) were identified as key genes, demonstrating consensus in terms of degree, betweenness, and maximum neighborhood component (MNC) network topological properties, and we are calling them key genes for our TNBC integrative analysis (Table 6).

**TABLE 5:** Topological analysis of PPI retrieved key genes.

Gene name	Degree	MNC	Betweenness
CDK1	60	60	18,044
STAT1	41	40	12,817
IL6	50	50	
PLK1	59	56	6782
CCNB1	52	52	9414
AURKA	44	44	8720
NDC80	46	46	3002
EGFR	72	65	56,082
IL1B	39	39	3744
FN1	40	39	14,678
BUB1B	50	50	2758

**TABLE 6:** Retrieved key genes.

Gene symbol	Description	Function
PLCG2	Phospholipase C gamma 2	Hydrolase, transducer
CXCL10	C‐X‐C motif chemokine ligand 10	Cytokine
CDK1	Cyclin dependent kinase 1	Control eukaryotic cell cycle (G2‐M, G1, and G1‐S)
STAT1	Signal transducer and activator of transcription 1	Activator, DNA‐binding
IL6	Interleukin 6	Cytokine, growth factor
PLK1	Polo like kinase 1	Kinase, transferase
CCNB1	Cyclin B1	
AURKA	Aurora kinase A	Cytokinesis, cell cycle progression
NDC80	NDC80 kinetochore complex component	Chromosome segregation, spindle checkpoint activity
EGFR	Epidermal growth factor receptor	Transferase, receptor, kinase
IL1B	Interleukin 1 beta	Cytokine, mitogen, pyrogen
FN1	Fibronectin 1	Heparin‐binding
BUB1B	BUB1 mitotic checkpoint serine/threonine kinase	Kinase, transferase

### 3.6 Regulatory Biomolecules of Breast Cancer

**FIGURE 7:**
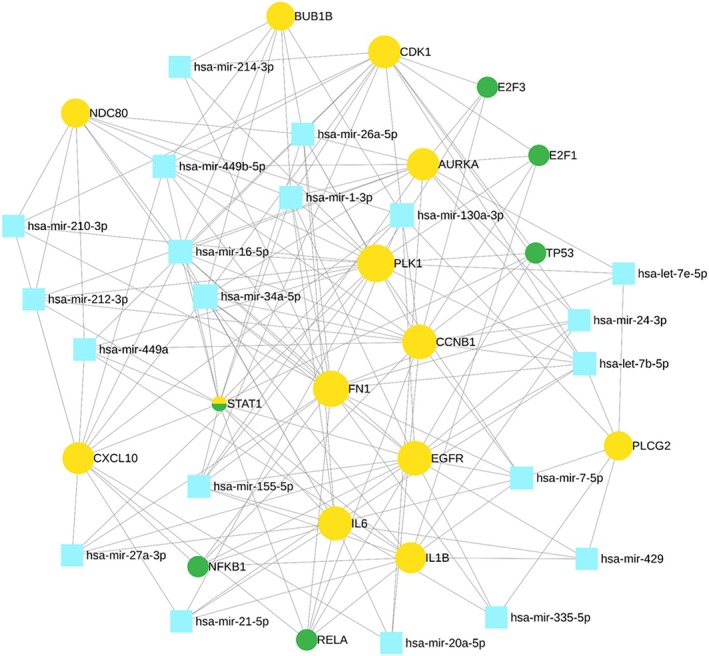
miRNA‐key gene‐transcription factor (TF) interaction network representation. Candidate genes are depicted in yellow circles, TFs in green circles, and miRNAs in sky blue squares. Utilizing the miRNet database, the network includes 20 miRNAs, 6 TFs, and 13 input genes as nodes, with 166 edges representing interactions. The network is generated based on topological properties, specifically when the degree > 3 applied on whole network.

We studied transcriptional and translational regulatory networks and identified 6 TFs (E2F3, E2F1, TP53, STAT1, NFKB1, and RELA) (Table 7) and 20 reporter miRNAs (Table 8) which showed significant values associated with key genes of TNBC (Figure 7).

**TABLE 7:** Identified TFs and their association with human disease.

TFs	Description	Associated with human disease
E2F3	E2F transcription factor 3	Dysregulated E2F3 has been identified associated with breast and other gynecological cancers [34]
E2F1	E2F transcription factor 1	Overexpressed E2F1 implication in cell cycle reported associated to gynecological and other cancers too [35]
TP53	Tumor protein P53	Mutation in TP53 gene found to be associated with early‐onset breast cancer other cancers too [36]
NFKB1	Nuclear factor kappa B subunit 1	NF‐kappaB pathway has been appeared to play a major role in inflammatory BC [37]
STAT1	Signal transducer and activator of transcription 1	Association of STAT1 in immune system alterations found contributed to the adult glioma [38]
RELA	RELA Proto‐oncogene, NF‐KB subunit	Upregulation of RELA has been identified as a key promoter of oral cancer progression, as well as other types of cancer [39]

**TABLE 8:** 20 miRNAs and their association in human disease.

miRNAs	Associated with human disease
hsa‐mir‐34a‐5p, hsa‐mir‐16‐5p, hsa‐mir‐1‐3p	Identified as key regulators in all the BC subtypes [40]
hsa‐mir‐130a‐3p	Discovered as potential post‐transcriptional regulators in TNBC [41]
hsa‐mir‐449b‐5p	Identified as potential biomarker for pancreatic and other types of cancer [42]
hsa‐let‐7b‐5p, hsa‐mir‐26a‐5p, hsa‐mir‐155‐5p	It has been found in breast tumor formation and progression [43, 44, 45]
hsa‐mir‐7‐5p, hsa‐mir‐449a	Identified as crucial regulators in various cancer subtypes, including lung cancer and other breast cancer subtypes [46]
hsa‐mir‐24‐3p, hsa‐mir‐212‐3p, hsa‐mir‐21‐5p, hsa‐mir‐210‐3p, hsa‐mir‐20a‐5p	It has been identified associated with liver diseases, epilepsy, and other subtypes of breast cancer [47, 48, 49, 50, 51]
hsa‐mir‐335‐5p, hsa‐mir‐27a‐3p, hsa‐mir‐429	It has been found associated with colorectal cancer and other cancers [52]
hsa‐let‐7e‐5p and hsa‐mir‐214‐3p	Identified as a potential biomarker for rectal carcinoma and thyroid [53, 54]

### 3.7 Cross‐Validation With TCGA

**FIGURE 8:**
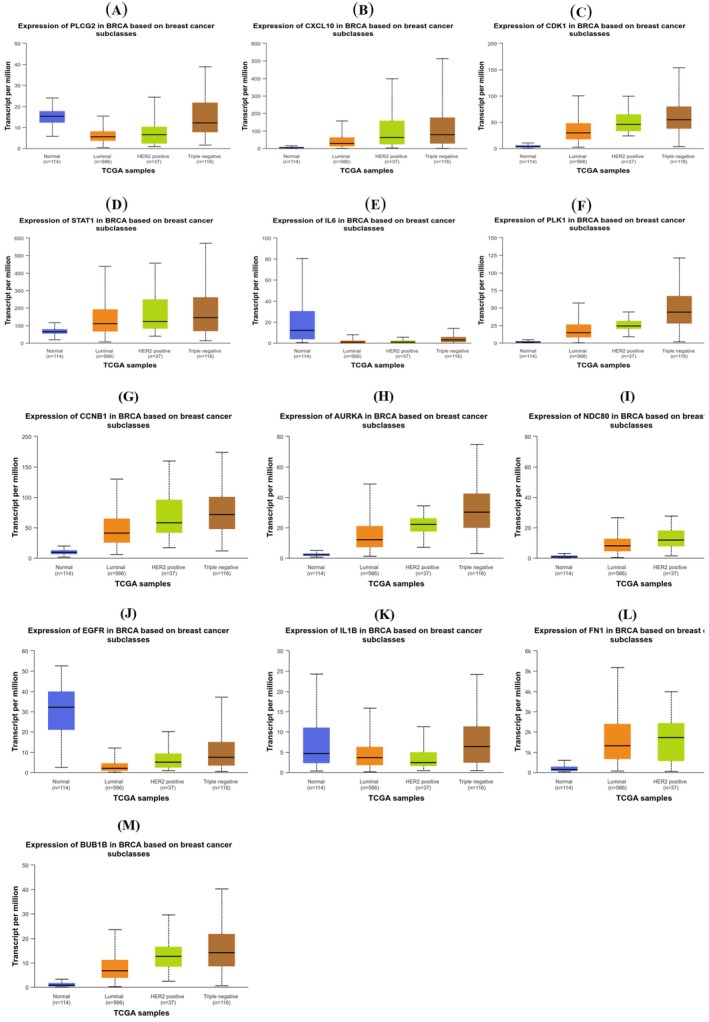
Cross validation of identified key genes. Box plots (A–M) representing the expression level of genes between normal and TNBC cases using UALCAN portal, data obtained from TCGA database.

The UALCAN integrative cancer data analysis portal (http://ualcan.path.uab.edu) was utilized to analyze the expression level of key genes in both normal and cancerous samples from patients with TNBC, data obtained from TCGA breast invasive carcinoma (BRCA). In total, there were 1211 samples, among which 114 samples were normal and 116 patient samples were categorized as TNBCs based on the immunohistochemical status of ER, PR, and HER2. Such manual curation identifies 10 differentially expressed key genes out of 13 that were found to be correctly dysregulated (Figure 8A–M). TPM (Transcripts per million) values for each gene in every sample were derived by multiplying the scaled estimate value by 1,000,000.

### 3.8 Cumulative Survival Analysis

**FIGURE 9:**
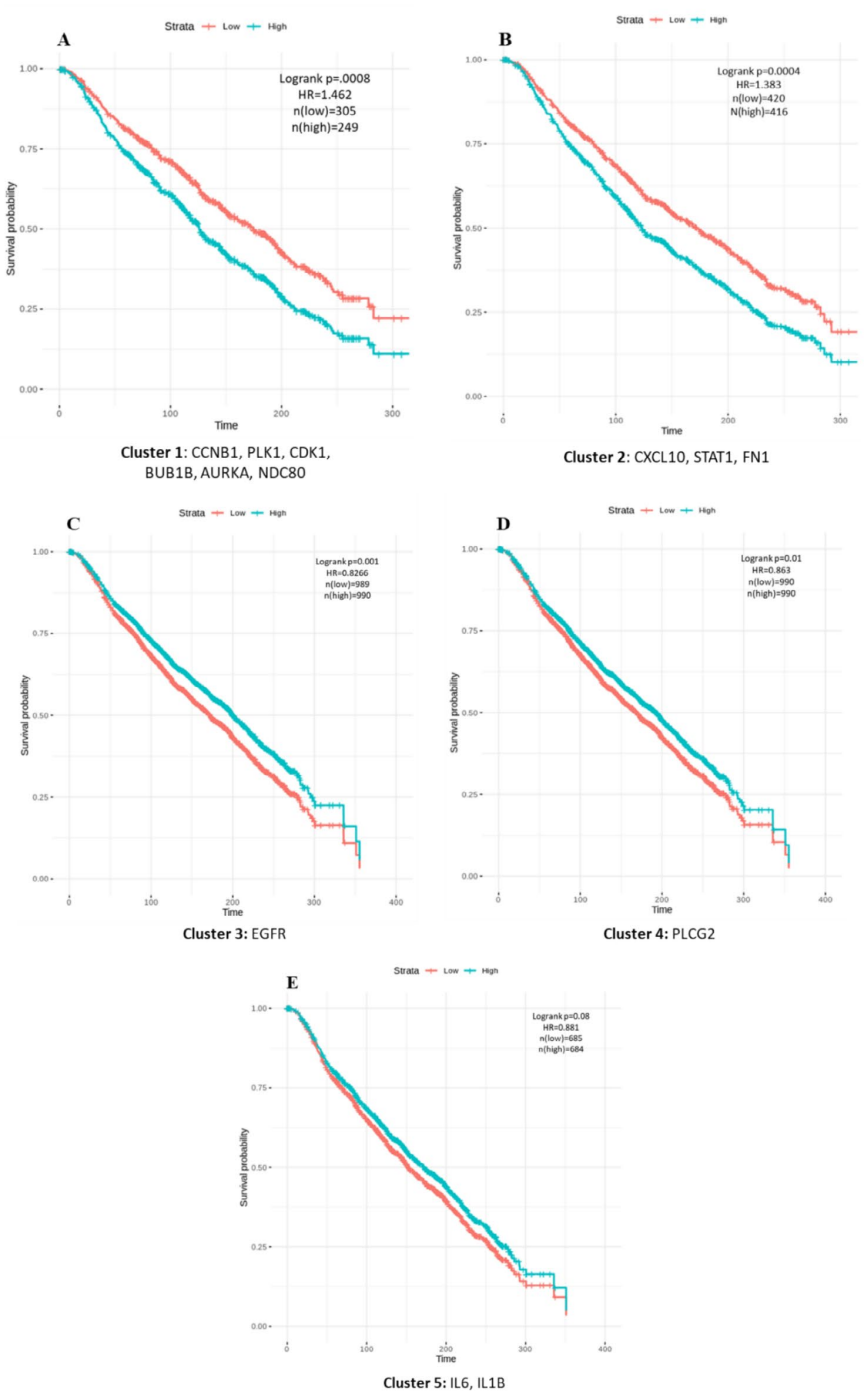
Cumulative survival analysis of retrieved key genes. (A–E) Survival curve KM plotted for each cluster and Log‐rank *p* < 0.05 were considered statistically significant.

To evaluate the collective impact of key genes on disease progression, at first, K‐means clustering was applied to group the key genes based on their expression into five clusters (Figure S2). To further assess the strength of relationships between genes in each cluster, Pearson's correlation analysis was conducted, and correlation plots were visualized for each cluster containing at least two genes (Figure S3). The survival outcomes for Cluster 1, which includes all the kinases, showed a *p*‐value of 0.0008 and a hazard ratio (HR) of 1.262. Cluster 2, consisting of growth factor genes, had a *p*‐value of 0.0004 and HR of 1.383. Clusters 3 and 4, each containing a single gene (EGFR and PLCG2), had *p*‐values of 0.001 and 0.01, with HRs of 0.8266 and 0.863. Cluster 5, consisting of interleukins, had a *p*‐value of 0.08 and HR of 0.881 (Figure 9A–E). This implies that over time, a high expression of kinases leads to a lower probability of survival with a significant hazard rate. A similar trend was observed in cluster 2, where a notable difference in survival was seen between low and high gene expression. However, clusters 3 and 4, which contain individual genes, and cluster 5, which contains two genes, do not exhibit such remarkable differences in survival based on their expression. We observed some significant statistical values when we performed survival analysis for individual genes in cluster 1. However, we did not obtain significant survival curves compared to the cumulative analysis (Figure S4). Thus, initially implemented on small‐scale datasets, this novel cumulative survival analysis could be a new therapeutic approach for large‐scale data to elucidate the impact of genetic complexity on patient survival.

## 4 Discussion

In recent years, there have been a large number of important studies on TNBC prevention, diagnosis, and treatment [2]. However, the molecular mechanisms regulating TNBC remain complex and poorly understood, and there are still lacking biomarkers for the early‐stage diagnosis of the disease [4]. The advancement of next‐generation sequencing platforms offers the identification of various molecular features of genes, including alternative gene‐spliced transcripts, post‐transcriptional modifications, gene fusions, mutations/single‐nucleotide polymorphisms (SNPs), and changes in the transcriptome [6, 7, 8]. Integrating RNA‐seq data from multiple platforms allows researchers to capture a broader spectrum of gene expression patterns, enhancing the robustness and accuracy of biomarker discovery [13]. WGCNA has emerged as a powerful tool for identifying modules of co‐expressed genes associated with clinical phenotypes [24]. By correlating module eigengenes or individual gene expression profiles with clinical phenotypes such as disease status, sex, age, condition, or treatment response, researchers can identify modules or individual genes that are significantly associated with the phenotype of interest [55]. PPI network analysis helps identify highly connected proteins (hubs) and densely interconnected protein clusters (modules) within the network [56, 57]. DEGs that are part of these hubs or modules may play critical roles in disease progression or other biological processes [58]. Therefore, through the integration of WGCNA and PPI analysis, we aimed to identify key genes that could serve as potential diagnostic and prognostic biomarkers for early‐stage disease diagnosis (Figure 1). In the DEGs analysis, we identified 2595 upregulated and 2001 downregulated genes (Table 2). Subsequently, the PPI network was reconstructed using these dysregulated genes, and to ensure a scale‐free network, topological properties were calculated (Figure 3 and Figure S1). Understanding the specific interactions between proteins and the formation of protein complexes is essential for advancing our knowledge of biological processes and developing new therapies for diseases [56, 57]. The PPI networks are also an essential tool for identifying new drug targets and developing new therapeutic strategies [59]. Within this network, modules of co‐expressed genes were identified, revealing potential functional clusters and key regulatory pathways associated with the observed gene expression changes. In the second bifurcated pipeline of WGCNA, we identified seven significant modules (Table 4) with correlation scores exceeding 0.90, each strongly associated with tumor phenotypic properties. A total of 13 key genes (Table 6) associated with TNBC were identified through consensus and integrated analysis, followed by PPI network reconstruction and hub gene (Table 5) identification of module genes retrieved through the two bifurcated pipelines. During the overlapping analysis, we identified two genes, PLCG2 and CXCL10 (Figure 6A), consistently found in both module identification methods. When we conducted an integrated analysis of module genes identified from both pipelines, followed by PPI and hub gene identification, we found a total of 11 genes, CDK1, STAT1, IL6, PLK1, CCNB1, AURKA, NDC80, EGFR, IL1B, FN1, and BUB1B (Figure 6B, Table 5), associated with TNBC. Hub proteins are those with the most connections and are required for the PPI network to function [58]. Computation of topological parameters such as Degree, Betweenness, and MNC in the above lines provides valuable insights into the optimal associations among edges and nodes within a network, elucidating the network's structure and identifying critical nodes with high centrality [38]. PLCG2, a crucial enzyme in transmembrane signaling [60], has been implicated in breast and other cancers [61], yet its specific association with TNBC remains unclear. CXCL10, involved in processes such as regulation of cell growth, differentiation, chemotaxis, and activation of peripheral immune cells, plays crucial roles in cancer‐specific pathways [62]. It has also been identified as a potential predictive biomarker for TNBC in other studies [34]. CCNB1, PLK1, CDK1, BUB1B, AURKA, and NDC80 are genes involved in various stages of the cell cycle process, which is crucial from a cancer perspective [35, 60]. These genes play significant roles in regulating cell division and are often dysregulated in cancer, making them potential targets for therapeutic intervention or biomarkers for diagnosis and prognosis. In other similar integrative bioinformatics studies, CCNB1, PLK1, CDK1, BUB1B, and AURKA have been recognized as potential hub genes [10, 11, 12]. IL‐6 and IL‐1β play diverse roles in biological functions, including immunity, tissue regeneration, and acting as potential pro‐inflammatory cytokines [36, 37]. The EGFR gene encodes a cell surface receptor involved in regulating cell growth, proliferation, and survival [39]. Activation by ligands triggers signaling pathways influencing cell division, migration, differentiation, and apoptosis [63]. Dysregulation or mutations in EGFR contribute to cancer development, making it a target for cancer therapies, including EGFR inhibitors [39, 63]. STAT1 regulates immune responses by activating genes involved in defense against pathogens and anti‐tumor immunity [64]. It also contributes to cellular differentiation, development, and homeostasis, but dysregulation can lead to autoimmune diseases, immunodeficiency disorders, and cancer [40, 41]. The FN1 gene encodes a glycoprotein crucial for cell adhesion, migration, tissue remodeling, and wound healing [42]. Its dysregulation is implicated in various pathological conditions, including cancer [43] and fibrosis [44]. Further, TFs and miRNAs were identified that control key genes at the transcriptional and translational levels associated with TNBC (Figure 7). TFs E2F3, E2F1, TP53, STAT1, NFKB1, and RELA (Table 7) were identified as dysregulated in nearly all gynecological cancers and several other malignancies [41, 45, 46, 47, 48, 49]. miRNAs a family of small non‐coding RNAs that regulate a wide array of biological processes, including carcinogenesis, are heavily dysregulated in cancer cells [50]. They can regulate breast cancer initiation and progression in different BC subtypes; therefore, they can be used as potential biomarkers [51]. In the current study, 20 reporter miRNAs (Table 8) were identified as significantly associated with BC. The hsa‐mir‐34a‐5p, hsa‐mir‐16‐5p, and hsa‐mir‐1‐3p miRNAs have been found to be key regulators in all the BC subtypes [52]. The hsa‐mir‐130a‐3p has been discovered to be post‐transcriptional regulators in TNBC [11]. The hsa‐mir‐449b‐5p has shown to be a potential biomarker for pancreatic and other types of cancer [53]. However, its relationship with TNBC and other subtypes of BC remains unexplored. The miRNAs hsa‐let‐7b‐5p, hsa‐mir‐26a‐5p, and hsa‐mir‐155‐5p have been implicated in breast tumor formation and progression; however, their specific role in TNBC remains to be investigated [54, 65, 66]. The hsa‐mir‐7‐5p and hsa‐mir‐449a miRNAs have been identified as crucial regulators in various cancer subtypes, including lung cancer and other BC subtypes [67]. The miRNAs hsa‐mir‐24‐3p, hsa‐mir‐212‐3p, hsa‐mir‐21‐5p, hsa‐mir‐210‐3p, and hsa‐mir‐20a‐5p have been linked to liver diseases, epilepsy, and other subtypes of breast cancer [68, 69, 70, 71, 72]. However, their potential association with TNBC needs to be explored. The hsa‐mir‐335‐5p, hsa‐mir‐27a‐3p, and hsa‐mir‐429 miRNAs have been found associated with colorectal cancer and other cancers [73]. The hsa‐let‐7e‐5p and hsa‐mir‐214‐3p have been implicated as a potential biomarkers for rectal carcinoma and thyroid [74, 75].

According to cumulative survival analysis (Figure 9A–E) the retrieved key genes have a high potential to be prognosticative biomarkers in TNBC. The Survival analysis, also known as time‐to‐event analysis, estimates the time it takes for a particular event to occur and provides tools to estimate the survival probability of patients over time. With advancements in high‐throughput sequencing techniques, gene expression data have become an invaluable resource in this field. This innovative cumulative survival analysis significantly broadens the scope of industrial applications by enabling a comprehensive assessment of survival outcomes associated with varying expressions of gene sets involved in critical biological pathways. This method surpasses traditional survival analysis, which typically evaluates the prognostic power of single genes over time, often falling short of fully understanding the mechanisms behind disease progression. This approach can identify multiple gene targets within disrupted pathways, facilitating the development of more effective drugs to improve survival rates. Consequently, this study offers the opportunity to explore significant biomarkers for TNBC in future research that can be validated with bench‐top experimentation.

## 5 Conclusion

Despite significant advancements in research to identify key genes and biomarkers for early detection, TNBC remains a challenging disease. Through this RNA‐seq integrative analysis, we have identified key genes significantly associated with TNBC, highlighting their relevance not only to women‐specific cancers but also to other cancer types. Further downstream analyses, including the identification of regulatory biomolecules such as TFs and miRNAs, collectively referred to as reporter biomolecules, as well as gene‐set enrichment, novel cumulative survival, and validation analyses, provide valuable diagnostic and prognostic insights. These findings suggest the potential therapeutic utility of these genes and their associated biomolecules. Thus, developing these biomolecules further for experimental research could result in a novel treatment for TNBC.

## Author Contributions

Pooja Singh and Pallavi Somvanshi acquired data, analyzed and interpreted data, and drafted a manuscript. Pooja Singh study concepts and design, data acquisition, analysis, and interpretation of data. Pallavi Somvanshi and Rupesh Chaturvedi have done data interpretation and manuscript drafting. All the authors reviewed and approved the manuscript.

## Ethics Statement

The authors have nothing to report.

## Consent

The authors have nothing to report.

## Conflicts of Interest

The authors declare no conflicts of interest.

## Supporting information


Data S1.
**Figure S1.** Topological analysis of PPI network.
**Figure S2.** Clustering analysis of the key genes.
**Figure S3.** Correlation analysis between the genes for each cluster.
**Figure S4.** Individual survival analysis for cluster 1 genes.

## Data Availability

The authors have nothing to report.
