# Supplementary material for: Network‐Based Integrative Analysis to Identify Key Genes and Corresponding Reporter Biomolecules for Triple‐Negative Breast Cancer
Source: Cancer Med. 2025 Apr 27;14(9):e70674. doi: 10.1002/cam4.70674 (PMC12034156; doi:10.1002/cam4.70674)
Supplement: Supplementary file 1 — Data S1. Figure S1. Topological analysis of PPI network. Figure S2. Clustering analysis of the key genes. Figure S3. Correlation analysis between the genes for each cluster. Figure S4. Individual survival analysis for cluster 1 genes. [file CAM4-14-e70674-s001.docx]

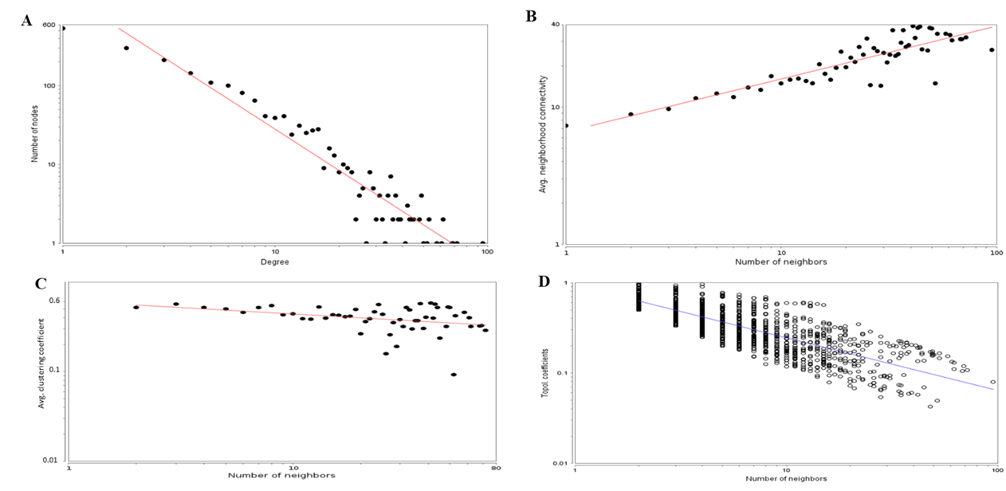


**Suppl. Figure 1. Topological analysis of PPI network.** **(A)** Degree distribution. The number of proteins with a given degree (k) in the TNBC PPI network follows a power law P(k) = ak^b^ (a = 1534, b = -1.737) and R^2^ = 0.906 for the power fit. **(B)** Average neighborhood connectivity distribution. Avg. neighborhood connectivity distribution was plotted against the number of neighbors. The neighborhood connectivity NC(k) with a given neighbor (k) in PPI network follows a power law NC(k) = ak^b^ (a = 6.638, b = 0.384) and R^2^ = 0.754 for the power fit. **(C)** Average clustering coefficient distribution. R^2^ = 0.143 for the power law fit. (D) Topological coefficient distribution. R^2^ = 0.652 for the power law fit.


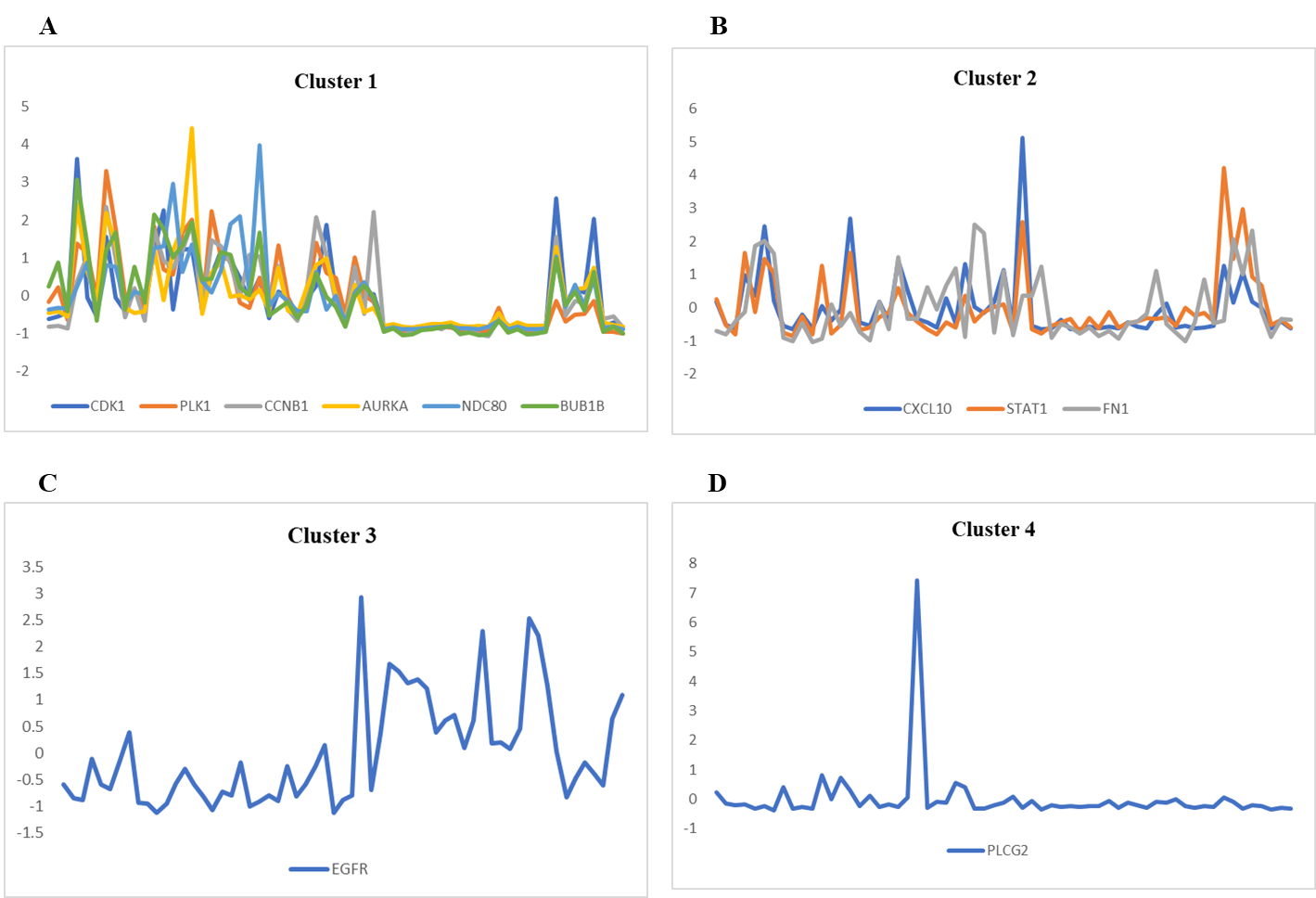


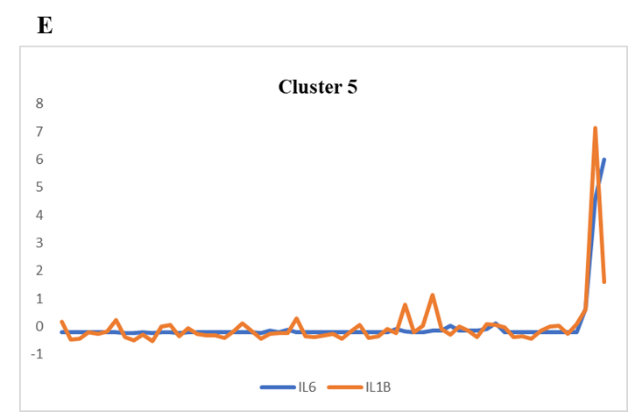


**Suppl. Figure 2.** **Clustering analysis of the retrieved key genes.** (**A-E**) To see the expression similarity of the key genes, K-means clustering was performed on the key genes with K = 5 clusters.


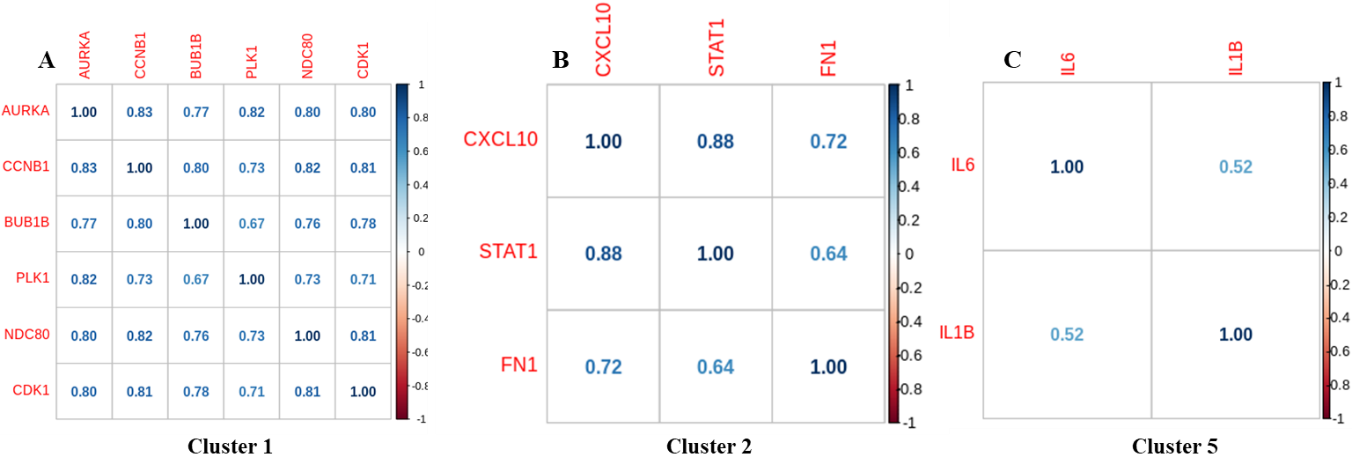


**Suppl. Figure 3. Correlation analysis between the genes for each cluster:** (**A-C**) To assess the strength of the relationships between the genes, Pearson’s correlation analysis was performed, and correlation plots were visualized for each cluster containing at least two genes.


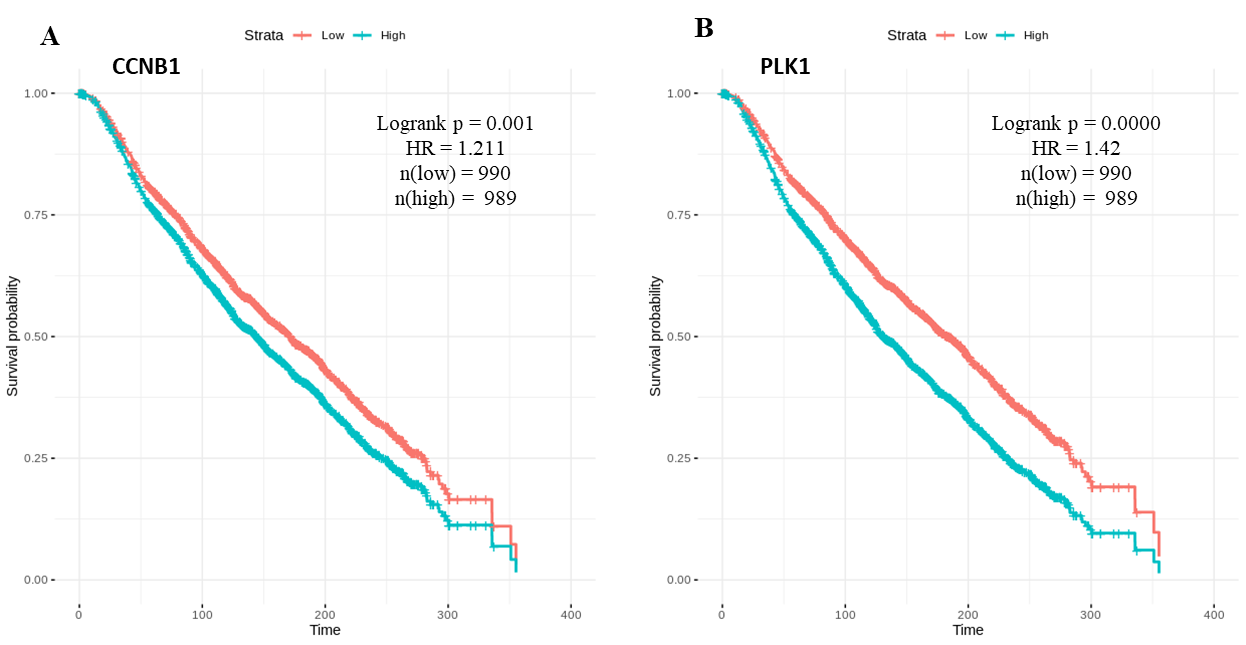


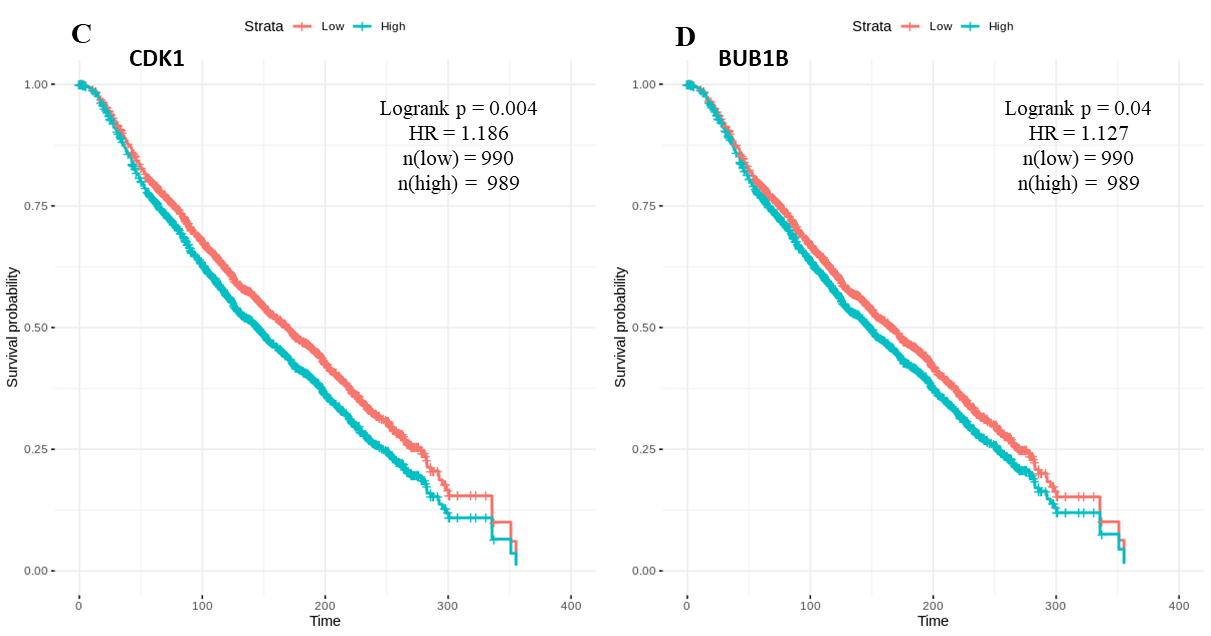


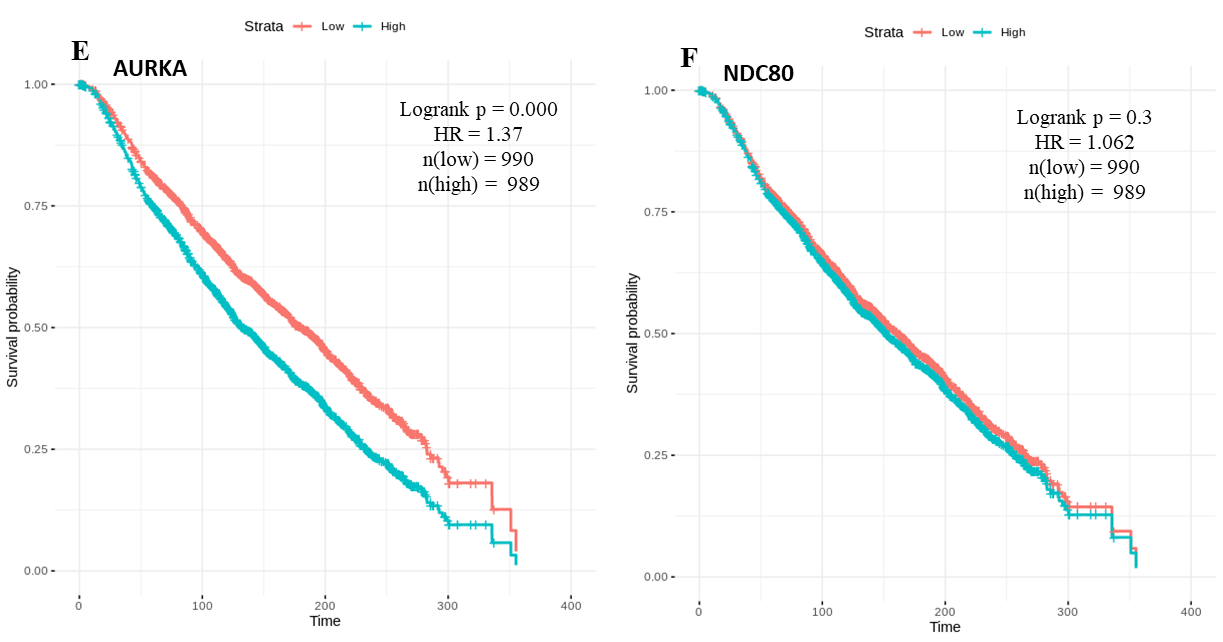


**Suppl. Figure 4.** Individual survival analysis for cluster 1 genes. (A-F) Survival curve KM plotted for each gene in cluster 1and Log-rank P value < 0.05 were considered statistically significant.
